# The Positive Effects of Trait Emotional Intelligence during a Performance Review Discussion – A Psychophysiological Study

**DOI:** 10.3389/fpsyg.2017.00463

**Published:** 2017-03-28

**Authors:** Mikko Salminen, Niklas Ravaja

**Affiliations:** ^1^Department of Information and Service Economy, Aalto University School of BusinessHelsinki, Finland; ^2^Helsinki Collegium for Advanced Studies, University of HelsinkiHelsinki, Finland; ^3^Helsinki Institute for Information Technology, Aalto UniversityHelsinki, Finland

**Keywords:** trait emotional intelligence, leadership, expressivity, social interaction, psychophysiology, emotion, EEG

## Abstract

Performance review discussions of real manager–subordinate pairs were examined in two studies to investigate the effects of trait emotional intelligence (EI) on dyad member’s felt and expressed emotions. Altogether there were 84 managers and 122 subordinates in two studies using 360 measured and self-reported trait EI. Facial electromyography, and frontal electroencephalography (EEG) asymmetry were collected continuously. Manager’s high trait EI was related to increased positive valence emotional facial expressions in the dyad during the discussions. The managers also had more EEG frontal asymmetry indicating approach motivation, than the subordinates. In addition, actor and partner effects and actor × partner interactions, and interactions between the role and actor or partner effect of trait EI were observed. Both actor and partner trait EI were related to more positive self-reported emotional valence. The results imply that trait EI has a role in organizational social interaction.

## Introduction

### Emotional Intelligence in the Workplace

Different definitions of emotional intelligence (EI) may differ greatly, but most of them share a common view of EI as a set of abilities that are related to emotions and emotional information. Salovey and Mayer (1990) were the first to introduce EI in the academic literature. Their ability model defined EI as a “subset of social intelligence that involves the *ability to monitor one’s own and others’ feelings and emotions, to discriminate among them and to use this information to guide one’s thinking and actions*” (Salovey and Mayer, 1990, p. 189). Trait models on the other hand define EI as “a constellation of emotion-related self-perceptions and dispositions located at the lower levels of personality” (Petrides et al., 2007, p. 26; Côté, 2014).

Different theories of EI thus conceptualize it, in varying degrees, either as a set of abilities or as traits; typically these are measured either by a maximum-performance test or by self-reports, respectively (e.g., Pérez et al., 2005). Trait EI can also be measured using informant approach, typically by 360-assessment (Conte, 2005). It has been suggested, that the difference between ability and trait EI is based on the measurement method and not on the types of components that the models putatively consist of Petrides (2011). In addition, studies utilizing both trait and ability EI measures have reported converging results, for example, a recent meta-analysis by Miao et al. (2016) found that both ability and self-report EI had a statistically significant contribution in predicting subordinate’s job satisfaction. There are, however, also contradictory views, which posit that the different streams of EI theories should be considered separately (e.g., Ashkanasy and Daus, 2005).

The strength of self-reports is their ease of use, with one of the drawbacks being social desirability bias in responding (e.g., Holtgraves, 2004). Ability tests of EI may seem to offer the most objective method, however, some concerns have been raised since, after all, there are no unambiguous criteria for determining correct responses in these types of tests (Pérez et al., 2005). One could argue that a strength of 360-method is that it provides feedback about the actual, real-life observed, EI and behavior. However, the obtained feedback depends heavily on the participants chosen for rating the person (typically a manager in an organization). This may be problematic, if the person being rated can freely choose the raters, and picks only those who view him favorably. It is thus advisable to use, if possible, different methods in conjunction when assessing EI. In the current two studies, we have used trait EI measured with 360 feedback, and self-reports.

During the last decades there has been a growing interest toward the possible positive effects of EI in organizational settings. Various studies have been conducted to gauge the extent of which the trait EI can explain in, for example, work outcome, leadership successfulness, organizational climate, or work wellbeing (e.g., Carmeli, 2003; Langhorn, 2004; Momeni, 2009; Martins et al., 2010). Effective emotional skills have also been linked to leadership emergence; it has been shown that good skills in perceiving, understanding and managing own and other’s emotions (measured with an ability EI test) is related to likelihood of attaining a leadership position in a small group (Côté et al., 2010). In a recent review, Côté (2014) summarizes several previous meta-studies and reviews that had considered various measures of EI and concluded that EI exhibits small correlations with several work criteria, including leadership emergence and value created and value claimed in negotiations. However, it is probable, as Côté (2014) notes that the effects of EI may depend also on the organizational context. For example, Joseph and Newman (2010) found, using meta-analytic data and a mixed model of EI, that EI predicts work performance positively in jobs that require emotional labor and negatively in jobs with less emotional labor. In addition, it has been shown that EI may not predict performance in individual cognitive tasks, but teams composed of high trait EI individuals score better in assignments than teams composed of low trait EI individuals (Jordan et al., 2002; Jordan and Troth, 2004). Thus, the possible positive outcomes of EI in work life are highly dependent on contextual factors.

EI as a concept and as a measure has received also a lot of criticism. It has been questioned whether the EI can predict leadership effectiveness beyond general intelligence or the big five personality dimensions (e.g., Antonakis, 2004). Most studies of the relationship between EI and work outcomes have not controlled enough these competing predictors of individual difference (Côté, 2014). In addition, there have been some attempts to link EI with transformational leadership (e.g., Gardner and Stough, 2002; Rubin et al., 2005; Harms and Credé, 2010), although this link has been questioned (Lindebaum and Cartwright, 2010). However, there are also studies, which suggest that at least the EI as a concept is not fully reducible to personality and IQ (e.g., Saklofske et al., 2003; Law et al., 2004) and a meta-analysis by O’Boyle et al. (2011) provided positive results about the EI’s role in work outcomes even when the personality and intelligence were controlled. The discussion about the validity of EI as a form of intelligence, and as a valid construct in studying organizational outcomes still continues. It may, after all, prove to be more fruitful to study manifestations of EI in actual leadership situations, for example, during social interaction between a leader and the followers. In the current study, the goal is to examine the role of trait EI in the felt and expressed emotions during a performance review discussion between a manager and a subordinate, using self-reports and psychophysiological measurements.

### Emotions, Social Interaction, and Psychophysiology

In the current study, emotional expressions of the participants were assessed by recording the activation of the facial muscles. During social interaction the expressive displays are affected not only by sociality of the context and the intensity of the emotion elicitor, but also by the relationship between the interacting partners (Hess et al., 1995). With the current two studies we focus to the communicative function of the facial expressions. Facial electromyography (EMG) has been utilized in previous studies as a signal of hedonic valence (see, for example, Cacioppo et al., 1986). Corrugator supercilii (brow) activity is related to negative emotional expressions and orbicularis oculi (periocular) activity has been related to positive high arousal emotional expressions (Witvliet and Vrana, 1995; Bradley and Lang, 2000). Orbicularis oculi is activated also during a, so-called, Duchenne smile (Ekman et al., 1990). Ekman and Friesen (1982) and Hess and Bourgeois (2010) note that smiles where the orbicularis oculi is activated are more reflective of the actual felt emotional state, whereas typically the so-called forced smiles are characterized by cheek muscle (zygomaticus major) activation and lack of orbicularis oculi activation. The facial EMG can be utilized also in the study of dynamics of emotional expressions during social interaction. For example, Mojzisch et al. (2006) studied the tendency to mimic facial gestures of other people during social interaction. This effect has been previously observed to happen also from people on video recording to the observers (Hsee et al., 1990).

Frontal asymmetry of the electroencephalography (EEG) is probably the most commonly used motivation and emotion-related EEG response (Coan and Allen, 2004). A consensus has been reached, according to which the anterior left and right brain areas would have central roles in the processing of approach and withdrawal motivations, respectively (Davidson, 2004). In addition, the approach-related emotions, such as joy and anger, are associated with relatively larger left frontal cortical activation, and withdrawal-related emotions, such as fear and disgust, are associated with relatively stronger right frontal activity (see, for example, Coan and Allen, 2003).

### Emotional Intelligence and Social Interaction

We chose a performance review discussion as the setting for organizational social interaction. During a performance review discussion it is possible that the manager may have to give also negative feedback, and thus the subordinate has to receive it. It is likely that emotional skills have an essential role in such a sensitive situation by helping to detect the emotional expressions of the partner and in controlling own emotional expressions. Since some of the facial expressions of emotion may be extremely subtle, the recording of the facial muscle activity was selected for the assessment of emotional expressivity.

Leadership can be defined as a process of influencing others to reach a goal (Yukl, 2010). Emotional expressions have a significant role in the practice of effective leadership; after all, face-to-face social interaction is an essential mechanism of influencing others. During social interaction overt and non-verbal cues work in combination to transmit information about emotions (Rashotte, 2002). In their review, Niedenthal and Brauer (2012) summed that facial expressions have a primary role in regulating social interaction and that accuracy of processing facial expressions, a mechanism close to one component of EI, according to several theorists (e.g., Salovey and Mayer, 1990), facilitates social interaction. Given the central role that emotions have in social interaction, it is not surprising that previous studies have established links between EI and social relations. High ability EI persons are perceived as more positively (reviewed in Mayer et al., 2008), and Schutte et al. (2001) found evidence for the links between trait EI and social skills and empathic perspective taking. Ability EI has been linked to positive relations with others, interpersonal sensitivity and prosocial tendencies, even when controlling for the effect of Big Five personality traits (Lopes et al., 2003, 2005). In a recent review, Petrides et al. (2016) concluded: “in general, high trait EI facilitates prosocial, and inhibits antisocial behavior.” We suggest that the manager’s high trait EI enhances social interaction by providing skills for detecting the partner’s emotional state from expressive behavior and by choosing own suitable emotional expressions. Thus, we propose the following hypothesis:

*H1*: The manager’s high trait EI leads to increased orbicularis and decreased corrugator activities during and more positively valenced self-reports after the performance review discussion in the dyad.

Negotiation is a typical situation of dyadic social interaction in an organizational context where the emotions play a significant role; some theorists have proposed that in negotiations emotions would be means of solving relational problems (Keltner and Haidt, 1999; Morris and Keltner, 2000). A performance review discussion can also be considered as a type of negotiation, where the participants are negotiating of, for example, a raise or a promotion. It has been shown that partner’s high ability EI is related to better outcome satisfaction in a negotiation (Müller and Curhan, 2006). Der Foo et al. (2004) found that high ability EI led to more positive experience in negotiation, but to lower objective gains; however, partner’s high ability EI led also to better objective gains. EI may function during negotiation in various ways; regulating one’s own emotions helps in keeping the interaction smooth and frictionless, observing partner’s emotions on the other hand helps in detecting subtle cues of the partner’s state, and expressing positive emotions can lead to reciprocation (e.g., Der Foo et al., 2004). During dyadic social interaction the EI of both of the persons has an effect to the outcome of the situation. It is suggested that high trait EI of both of the participants of a dyad leads to both, subjectively experienced and expressed, positive emotions. Thus, we propose the following hypothesis:

*H2*: Actor and partner high trait EI lead to increased orbicularis and decreased corrugator activities during and more positively valenced self-reports after the performance review discussion between the manager and the subordinate.

### Emotional Expressiveness

Emotional expressiveness, characterized by, e.g., eye contact, effective facial expressions, and vocal variety, has been linked to perceptions of likability during initial interactions (Friedman et al., 1988). Expressiveness has been studied previously, for example, in the context of visionary and charismatic leadership. Groves (2006) concludes on the relationship between EI and visionary leadership that visionary leaders are skilled in communicating a compelling and inspiring vision to the followers. George (2000), on the other hand, relates to EI the leader’s ability to motivate followers, by evoking enthusiasm and trust.

Charismatic leadership is conceptually close to visionary leadership (e.g., Conger and Kanungo, 1987). In contrary to rational, transactional leadership, transformational and charismatic leadership theories put more emphasis to emotional and inspirational leadership influence and to charismatic communication style of the leader (Bono and Ilies, 2006). The positive effects of charismatic leadership on performance and motivation have been highlighted in a meta-analysis by Judge and Piccolo (2004). Some researchers have linked charismatic leadership to, not only emotional expressiveness, but also to other emotional skills, such as emotional expression regulation and sensitivity to follower’s emotions (Conger and Kanungo, 1998; Riggio and Reichard, 2008), which suggests a close relationship between charismatic leadership and EI. Charismatic leaders are typically described as having special personal qualities that enable them to exert extraordinary influence on the followers (Conger and Kanungo, 1987). Some of the mechanisms of influencing are suggested to be related to emotional skills. For example, Bono and Ilies (2006) found leader’s positive emotional expressions to be related to ratings of their charisma in a real-life organizational setting. Considering previous studies, there is resemblance between skills linked to EI and emotional skills attributed to visionary and charismatic leadership. In addition, visionary and charismatic leadership are related to emotional expressivity. It is suggested that the high trait EI participants would be more expressive of both positive and negative valence emotions, as indexed by facial EMG activation; thus, following hypothesis is presented:

*H3a*: High trait EI is related to increased emotional expressiveness, as indexed by facial-EMG activation on orbicularis oculi and corrugator supercilii sites.

The manager’s emotional expressions are an important source of information for the other members of the organization; it has been suggested that followers of leaders with positive affect could carry the contagious positivity to their daily encounters with the clients which would result to, for example, better sales numbers (George and Bettenhausen, 1990; van Kleef, 2014). The status difference between the manager and the subordinate is suggested to have an effect to the emotional expressivity. Approach/Inhibition Theory of Power (Keltner et al., 2003) posits that individuals with higher power feel more approach vs. inhibition emotions. It must be noted, that not all approach emotions are positive, anger is an example of a negative approach emotion. In addition, the Expressivity Demand Theory (LaFrance and Hecht, 2000; Hess and Bourgeois, 2010) states that individuals with higher power have more freedom in choosing their emotional expressions, whether positive or negative. Thus, we suggest that the leaders are more expressive, and propose the following hypothesis:

*H3b*: Leaders have more EMG activity on orbicularis oculi and corrugator supercilii facial muscle areas than followers.

Previous studies have linked high trait EI with resting-state left frontal activation (e.g., Mikolajczak et al., 2010). High ability EI has also been linked to ratings of socially engaging behavior during dyadic interaction (Brackett et al., 2006). Thus, we suggest the following hypothesis:

*H4*: High EI is related to approach motivation as indexed by the EEG frontal asymmetry.

## Current Studies

Even though the positive effects of the EI on social interaction are documented in various studies, the actual expressive behaviors by which these effects are manifested during social interaction have not received the same amount of attention. In the current two studies, the psychophysiological methods are used to investigate facial expressive behaviors, namely smiling and frowning.

Building upon the work on negotiation by Der Foo et al. (2004), we suggest that high trait EI assists both the manager and the subordinate in conducting a performance review discussion, by helping in controlling one’s own emotions, detecting emotions of the partner, and expressing own emotions appropriately. We follow the suggestions of Morris and Keltner (2000) in studying particular forms of emotional expression, smiling and frowning, during dyadic interaction between a manager and a subordinate. The topic of the current study, non-verbal communication of emotions in organizational setting, has also practical value, given that a study by Newcombe and Ashkanasy (2002) suggested that leader’s non-verbal emotional cues have a strong effect on the member’s ratings of the quality of leader–member relationship.

The hypotheses presented above were studied with two settings. The goal of the first study was to examine the hypotheses 1 and 3a and to study the effects of the leader’s trait EI on the self-reported emotions and psychophysiological activity of the dyad. For ecological validity we chose to let the participant’s conduct the performance review discussions following the guidelines of their own organizations and thus, the duration of the discussions was not controlled either. Each leader had a discussion with two followers and facial EMG was collected from all of the participants. The leader’s trait EI was assessed using a 360 questionnaire of EI, for obtaining an assessment of the leader’s trait EI as observed by his or her colleagues during every day work-life encounters.

For the second study, the main goal was to examine the hypotheses 2, 3b, and 4. We chose not to use the actual performance review discussion guidelines and forms of the companies, because in the first study this had led the discussants to follow them too strictly, to the cost of a more natural and dialogical interaction. Instead, we presented the leader and the follower with an instruction that encouraged more equal dialog. In addition, we decided to limit the duration of the discussion to encourage the participants to stay on topic. The methodology for the second study changed; EEG was collected from the participants to assess approach and withdrawal motivations and, due to the added time for placing the electrode caps, only one discussion for each leader was recorded. Both, the subordinates and the leaders, filled self-reported trait EI questionnaires. Thus it was possible to study actor and partner effects of trait EI using the methodology presented by Kenny et al. (2006).

## Study 1

### Participants

The participants were 40 managers (24 female) and 78 (45 female) of their subordinates from five different public or private sector organizations from various fields (e.g., education, banking, media, chemical engineering). Each manager had discussions with two of her own subordinates, with the exception of two managers who had, due to scheduling problems, discussion with only a one subordinate. The mean age was 44.5 (*SD* = 8.9) years for the managers and 45.5 (*SD* = 9.9) years for the subordinates. In the recruitment process the managers were contacted first and they were asked to recruit two of their own subordinates. In addition, the order of the discussions with the two subordinates was decided by the managers. This study was carried out in accordance with the recommendations of the Finnish Advisory Board on Ethical Integrity. The study was not medical research. Thus, the need for formal approval was not required, at the time of conduct, by the ethics review board of our university. All subjects gave written informed consent in accordance with the Declaration of Helsinki.

### Setting/Procedure

The whole measurement session lasted approximately 3 h, consisting of informing the participants about the forthcoming session, filling consent forms, attaching the electrodes and preparing the recording devices, the baseline session, the actual discussion, filling in self-report forms, removing the electrodes, and responding any additional questions. The manager and the first of the subordinates were attached to the measurement devices and this was followed by a 5-min baseline recording. After this video cameras were turned on and the participants were free to discuss as long as needed. The manager was asked to perform a typical performance review discussion, following the structure and guidelines of the organization. All the measurements were conducted at the premises of the participating companies, either in a meeting room or in the manager’s own office room. The discussing partners were seated by a table, and a stand for two web-cameras was placed in between them for collecting feedback material for the participants to process during a leadership development program. Additional back-up video-cameras were placed next to each participant. The researchers waited in an adjacent room or in the hallway during all the recordings.

After the first discussion, the manager could take a short rest and prepare for the second discussion as the second subordinate was being attached to the measurement device. On average the discussions lasted for 80 min (*SD* = 28 min) with maximum duration of 2 h 48 min and minimum duration 42 min.

### Questionnaires

After the discussion, the manager and the subordinate filled a questionnaire assessing their emotions during the discussion. Self-ratings of emotional valence were collected with 9-point graphical scales that resemble Lang’s (1980) self-assessment manikin (SAM). A paper version of the SAM scale was used. The SAM Valence scale was composed of nine graphic representations of a manikin with facial expressions ranging from a smiling and happy to frowning and unhappy. The SAM Arousal scale ranged from an excited and wide-eyed figure to a relaxed and sleepy figure (see, for example, Bradley and Lang, 1994). The SAM scales have been widely used in various settings, for example, with pictorial stimuli (Backs et al., 2005), auditory stimuli (Siegert et al., 2011), in applied fields such as advertising studies (Morris, 1995), and also in the study of social processes (e.g., Kirsch et al., 2005; Coan et al., 2006; Kolassa and Miltner, 2006; Buckner et al., 2010). The validity of the SAM Valence and Arousal scales were established in a study by Bradley and Lang (1994) which reported significant correlations with the Semantic Differential Scale.

For each manager a 360-degree feedback of their trait EI was assessed using the Finnish EI360 questionnaire (Saarinen, 2007; Salminen et al., 2013) that consisted of 37 statements, which were rated using a 5-point scale (1 = does not describe the person at all, 5 = describes the person very well). The questionnaire was originally designed to consist of seven factors: (1) Perceiving emotions (four statements, e.g., “He recognizes when someone is sad or depressed”), (2) Openness of emotional expression (six statements, e.g., “He willingly describes his emotions to others”), (3) Expression of negative emotions (five items, e.g., “He expresses when he is getting nervous”), (4) Emotional facilitation (six items, e.g., “He uses strong and emotionally appealing arguments”), (5) Understanding emotions (four items, e.g., “He understands how mood affects the behavior of people”), (6) Regulation of emotions (five items, e.g., “He can deal with uncertain circumstances”), and (7) Using emotions (seven items, e.g., “He gets along well with other people”). The intra-class correlations for the seven factors were sufficient (0.912, 0.902, 0.901, 0.886, 0.953, 0.873, and 0.935, respectively), thus, for the current study an overall score of trait EI was used. Feedback was requested from the managers themselves, their subordinates, their peers, and their own managers. On average, 5.8 persons gave ratings for each manager, most being their subordinates. In the analyses reported here, we have omitted the self-reported ratings of the managers and have used a mean of ratings provided by others. The alpha reliability for the total score of the EI360 questionnaire in the current sample was 0.83.

### Psychophysiological Recordings

The physiological signals were recorded using two Varioport-B portable recording systems (Becker Meditec, Karlsruhe, Germany). Facial EMG activity was recorded from the left corrugator supercilii and orbicularis oculi muscle regions, as recommended by Fridlund and Cacioppo (1986), using surface Ag/AgCl electrodes with a contact area of 4 mm diameter (Becker Meditec, Karlsruhe, Germany). Electrodes were filled with Synapse conductive electrode cream (Med Tek/Synapse, Arcadia, CA, USA). The raw EMG signal was sampled at 1024 Hz, amplified, and frequencies below 57 Hz and above 390 Hz were filtered out, using the analog filter built in the Varioport device. The recording device was attached with a carrying belt to each participant’s chest. All data were stored on a CompactFlash memory card (2 GB) after digitizing with a 16-bit A/D converter.

In addition, electrodes were placed to left zygomaticus major muscle area, but due to the artifacts caused by speech actions the results of this signal is not reported here.

### Data Reduction and Preprocessing

Data were analyzed using Matlab (v.7.7.0) software with Anslab Professional (v.2.4) toolbox. The EMG signals were rectified and smoothed using a 50 ms moving average. The processed signals were saved using a 10 Hz sampling rate. Mean values of the facial EMG signals for the whole discussion duration were calculated. Finally, these values were natural log-transformed to normalize the distributions.

### Statistical Analyses

The data were analyzed by the linear mixed-models procedure in SPSS with restricted maximum likelihood estimation and compound symmetry: heterogeneous covariance structure for the residuals. In the model, the running number of discussion was set as the subject variable and role (manager/subordinate) × order of discussion for the manager (1st/2nd) was set as the repeated variable. The role, order of discussion for the manager, number of females in dyad (none, 1, or 2) and the 360-assessed manager’s trait EI and it’s interaction with the role were included in the model. When testing for the psychophysiological mean values, the mean of respective baseline value was also included in the model.

### Results

The results of the statistical tests are presented in **Table 1**.

**Table 1:** Results of the statistical tests for Study 1.

Source	*df*	*F*	*p*
**Gender**			
Valence rating	2, 57.94	9.12	<0.001
**Manager’s 360-assessed emotional intelligence**			
OO	1, 52.627	5.68	0.021


*Only statistically significant results are included. OO, orbicularis oculi.*

The manager’s EI had a statistically significant main effect on the dyad member’s orbicularis oculi activities, *p* = 0.021. Manager’s high EI was related to higher Δ (mean activity during the discussion - mean activity during the baseline) orbicularis (*M* = 0.336, *SD* = 0.566) activity than manager’s low EI (*M* = 0.175, *SD* = 0.646).

In self-reported emotional valence after the discussion there was a statistically significant effect of gender, *p* < 0.001; the participants reported more positive emotional valence in a female–female dyad (*M* = 7.28, *SD* = 0.88) when compared to a male–male (*M* = 6.53, *SD* = 1.14), or to a female–male dyad (*M* = 6.34, *SD* = 1.34).

### Brief Discussion

Manager’s high trait EI was related to more orbicularis oculi activity in the dyad during the discussion. This result suggests that the manager’s trait EI evoked more positive valence emotional facial expressions in the participants, thus supporting the Hypothesis 1. In addition, the facial EMG result suggests that trait EI is positively related to emotional expressiveness, shown as increased positive valence emotional expressions measured on the orbicularis oculi site (Hypothesis 3a). Also, the gender of the dyad members had an effect to the self-reported emotions; most positive valence and happiness was rated in all-female dyads.

## Study 2

### Participants

Forty-four manager–subordinate dyads from eight different private organizations from various fields (e.g., food production, media, social services, engineering, daily consumer goods trade, cleaning, and facility services) participated to the study. Mean age of the managers was 43.0 years (*SD* = 8.5) and of the subordinates 41.9 (*SD* = 9.0). There were 18 female managers and 24 female subordinates. Sixteen of the dyads were male–male, 14 were female–female, and 14 were mixed gender dyads. The managers were free to choose a willing subordinate for the discussion. None of the participants of the Study 1 participated to the Study 2. This study was carried out in accordance with the recommendations of the Finnish Advisory Board on Ethical Integrity. The study was not medical research. Thus, the need for formal approval was not required, at the time of conduct, by the ethics review board of our university. All subjects gave written informed consent in accordance with the Declaration of Helsinki.

### Setting

The setting was similar with Study 1 with the following changes: (1) Each manager had discussion with only one of his or her subordinates. (2) There was a 30-min time limit for the discussion. (3) Few days prior to the discussion the participants were sent a message with a request to beforehand think of three developmental issues for themselves and for their discussion partner. The participants were asked to discuss these topics in a free order during the recorded discussion.

### Questionnaires

After the discussion, the manager and the subordinate filled a questionnaire assessing their emotional state during the discussion. Ratings of emotional valence were collected with 9-point graphical scales that resembled Lang’s (1980) self-assessment manikin, following the procedures of Study 1.

Both, manager and subordinate, filled also self-reported 33-item EI questionnaire, developed by Schutte et al. (1998). The form was translated in 2000 to Finnish by the Jyväskylä University, Human Development and Risk Factors Program. The alpha reliability for the total score was 0.86.

### Psychophysiological Recordings

The setup for the recording devices was similar with Study 1, with the EEG added. EEG was recorded from six sites (F3, F4, C3, C4, P3, P4) using linked mastoids as the reference. The Ag/AgCl electrodes were placed on a stretch cap following the international 10/20 system. In addition, electro-oculogram was measured to detect vertical and horizontal eye-movements to facilitate the removing of eye-movement related artifacts; the bipolar electrodes were placed above and below of the right eye and to the outer canthi of both eyes, respectively. The EEG signal was sampled with 256 Hz rate using 0.1 Hz high pass and 100 Hz low pass filters.

### Data Reduction and Preprocessing

The EMG data was analyzed using Matlab (version R2012b) software and Signal Processing and Statistics toolboxes. A notch filter at 50 Hz and a high-pass filter at 90 Hz were applied, in addition the signals were rectified and smoothed with a 100-ms moving average window.

The EEG signal was analyzed with Analyzer 2 software (Brain Products Inc.). The eye-movement artifacts were removed by using an ocular correction algorithm (Gratton et al., 1983). In addition, those 1-s segments that contained activity exceeding ±85 μV were removed. For the remaining epochs, the power spectra were derived using the fast Fourier transform with Hanning window (applied to the distal 10% at the ends of the epoch). For each epoch power values (μV^2^) from the alpha band (8–12 Hz) were extracted. A frontal asymmetry index was calculated, using natural logarithmic transformation, with an equation ln(F4) - ln(F3) with higher scores indicating greater relative left frontal activity (e.g., Allen et al., 2004). Mean values of all of the described signals were calculated for the 5-min baseline and for the 30-min discussion, and all values were log-transformed to normalize the distributions.

### Statistical Analyses

Actor–partner interdependence model, formulated by Kenny et al. (2006), with linear mixed models in IBM SPSS Statistics (version 20), was used. The dyad members, a manager and a subordinate, had distinguishable roles. Prior to analyses all the values of the predictor variables were centered around their dyad means as suggested by Kenny et al. (2006). In the model each participant served as both an actor and a partner. An actor effect is the effect that a predictor variable has on that same person’s outcome variable, whereas a partner effect is the effect that a person’s predictor variable has on his partner’s outcome variable (Kenny et al., 2006, p. 145).

In the model, dyad was set as the subject variable and role (manager/subordinate) as the repeated variable and compound symmetry: heterogeneous was used as the covariance structure. Actor and partner effects of EI, and their interactions, were included in the model; also the number of females in the dyad was included in the model. In addition, role and interactions of role with the actor and partner effects of EI were also included in the model. When testing for the psychophysiological values the respective baseline value was included in the model.

### Results

The results of the statistical tests are presented in **Table 2**.

**Table 2:** Results of the statistical tests for Study 2.

Source	*df*	*F*	*p*
**Gender**			
CS	2,23.69	3.48	0.047
EEG frontal asymmetry	2,20.70	3.83	0.038
**Role**			
EEG frontal asymmetry	1,26.645	5.13	0.032
**Self-reported emotional intelligence**			
Valence rating			
Actor	1,45.75	10.62	0.002
Partner	1,44.35	12.31	0.001
Actor × partner	1,29.57	4.75	0.037
EEG frontal asymmetry			
Role × actor	1,34.33	6.47	0.016


*Only statistically significant results are included. EEG, electroencephalography; CS, corrugator supercilii; Role, manager/subordinate.*

For gender, there were two statistically significant main effects on corrugator supercilii activity, *p* = 0.047 and on EEG frontal asymmetry, *p* = 0.038. There was higher Δ corrugator activity in female–female dyads (*M* = 0.53, *SD* = 0.52) than in female–male (*M* = 0.47, *SD* = 0.49), or in male–male (*M* = 0.38, *SD* = 0.56) dyads. In frontal asymmetry relatively highest left frontal activity, suggesting approach motivation, was observed in male–male dyads (*M* = 0.0016, *SD* = 0.19), compared to female–male (*M* = -0.016, *SD* = 0.18) or female–female (*M* = -0.038, *SD* = 0.24) dyads.

There was also a statistically significant main effect for role on frontal asymmetry, *p* = 0.032; there was relatively more left frontal activity, suggesting approach motivation, on managers (*M* = -0.01, *SD* = 0.24) than on subordinates (*M* = -0.02, *SD* = 0.15).

**FIGURE 1:**
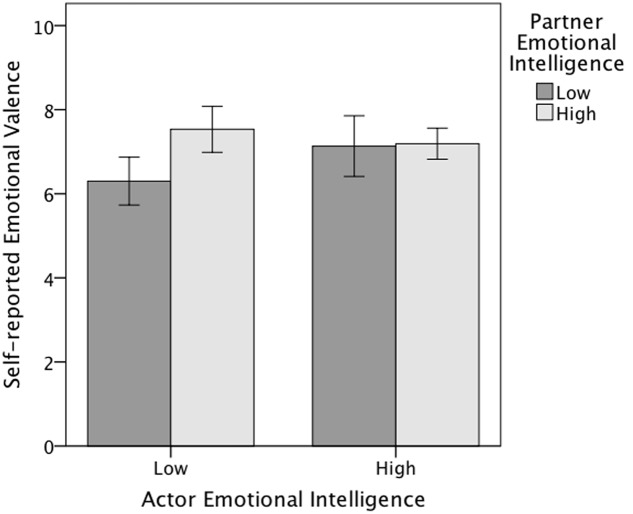
**Actor × partner interaction effect of trait emotional intelligence on the self-reported emotional valence (Study 2).** Error bars represent 95% confidence intervals.

In the emotional valence ratings there were statistically significant actor, *p* = 0.002, and partner, *p* = 0.003, effects of trait EI. Actor high trait EI was associated with more positive self-reported valence (*M* = 7.05, *SD* = 1.14) than actor low trait EI (*M* = 6.88, *SD* = 1.22). Similarly, partner’s high trait EI was associated with more positive self-reported valence (*M* = 7.29, *SD* = 0.90) than partner’s low trait EI (*M* = 6.64, *SD* = 1.33). The positive effect of partner’s high trait EI was more prominent for actors with low EI than for actors with high EI; thus there was also a statistically significant actor × partner interaction effect, *p* = 0.037 (**Figure 1**) on the rating of emotional valence.

**FIGURE 2:**
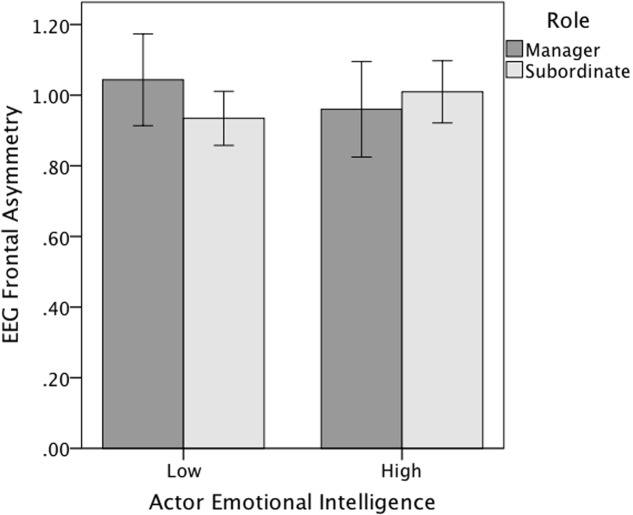
**Role × actor trait emotional intelligence interaction effect on the EEG frontal asymmetry (Study 2).** Error bars represent 95% confidence intervals.

In addition, there was a statistically significant interaction effect between role and actor effect of trait EI on the EEG frontal asymmetry, *p* = 0.016. For managers, low trait EI was associated with more frontal asymmetry suggesting approach motivation than high trait EI. However, this was opposite for the subordinates; for them high trait EI was associated with more frontal asymmetry suggesting approach motivation than low trait EI (**Figure 2**).

### Brief Discussion

There was most corrugator supercilii activity, indicating, negative emotional expressions in all-female dyads; this result is contrary to Study 1, and this discrepancy should be examined more thoroughly in future studies. The observation that there was most approach-related EEG asymmetry in all-male dyads, and least in all-female dyads, are in line with the corrugator findings. The role had also an effect to the observed frontal asymmetry during the discussion; the managers had more EEG-asymmetry-measured approach motivation, possibly because they were more strongly involved with the discussion. After all, the managers probably felt that they were more responsible for conducting the discussion and that their performance was more under scrutiny. An interaction result indicated that there was more approach motivation for low trait EI managers and vice versa for the subordinates, thus the Hypothesis 4 was only partially confirmed. It is suggested that this was due to the low trait EI managers trying to compensate their lack of emotional skills by increased engagement and activity. Contrary to the Hypothesis 3b, there was no role difference in the expressivity.

For the valence ratings there were actor and partner effects, as well as an interaction of these. Both actor and partner trait EI led to more positive valence ratings, confirming the Hypothesis 2. Also, if the own (actor) trait EI was low the partner’s high trait EI had a more positive effect on the rating of valence.

## General Discussion

The current studies examined the effects of trait EI on self-reported emotions and physiologically measured emotional expressivity during organizational dyadic interaction. The aim was to clarify the actual expressive patterns, which manifest trait EI in social interaction between the subordinate and the manager.

The findings provide support to the suggestion that both actor and partner trait EI lead to more positive subjective perceptions of dyadic social interaction. Prior studies on the effects of EI in face-to-face social interaction have seldom reported analyses of actor and partner effects. In a study by Sy et al. (2006), although not reporting actor and partner effects *per se*, found that employee’s trait EI was linked positively to their performance and job satisfaction, and also the manager’s EI had a more positive correlation with low EI employee’s job satisfaction than with high EI employee’s job satisfaction. The authors suggested that the low EI employees benefit more of the manager’s help in regulating their own emotions, whereas the high EI employees more probably already experienced higher levels of job satisfaction, and they were also more adept in managing their own (negative) emotions. Somewhat similarly, in the current study we observed that the positive effect of partner’s high trait EI on self-reported emotional valence was more prominent for low trait EI actors than for high trait EI actors. It is suggested that the low trait EI actors benefited more of the high trait EI partner’s ability to keep the emotional tone of the discussion positive.

The obtained results were not unequivocal for the suggestion that trait EI would be related to increased emotional expressivity. This suggestion held true for the positive emotional expressions, but not for the negative emotions. Previous studies have highlighted the indisputably favorable effects of positive emotions in organizational settings. Besides the effect that positive emotions have on individual performance, such as increased motivation, performance, cooperation (Bono and Ilies, 2006), and creativity (Isen, 2004), positive emotions have a strong effect in organizational social interaction. Negative emotions expressed by the leader may act as a warning signal to the followers, indicating that the leader may not be fully in charge of events. The detrimental effects of leader’s expression of negative emotions was shown in an experiment by Gaddis et al. (2004), where positive emotions expressed during negative, failure-related, feedback were related to higher perceived effectiveness of the leader than expression of negative emotions in a study using undergraduate students in a group-work setting (Gaddis et al., 2004). van Kleef (2014) reviews previous studies on the topic and notes that leader’s positive emotional expressions are related to ratings of better leadership quality by the followers (Lewis, 2000). The effect of leader’s mood contagion to the team was shown by Sy et al. (2005), leader’s positive mood was contagious to the moods of the team members and lead to increased coordination, while the leader’s negative mood was related to increased effort in the team (Sy et al., 2005).

Besides using positive emotional expressions in building, e.g., trust and motivation, the suppression, or controlling of emotional expressions is also a relevant skill in work settings (e.g., Gross, 1998) and a mechanism of impression management. Although in general the expression of negative emotions is discouraged (Staw et al., 1994; Brotheridge and Gandey, 2002), there are, however, many situations in work life, where the leader would have to give negative feedback, possibly accompanied by negative emotional expressions. A performance review discussion is potentially such an event, where the leader must, if needed, give negative feedback on the follower’s productivity, behavior, or other issues. Effective skills in expression and control of emotions would help the leader to communicate to the follower his or her areas of development so that the atmosphere would be constructive (Riggio and Reichard, 2008). Lindebaum and Fielden (2011) reported of an example of how negative emotions can be used deliberately; they found that leaders in the construction business expressed anger to make sure that projects would be finished in time. This energizing effect of negative emotions could, in the hands of an emotionally intelligent leader, turn out to be a powerful tool. Negative emotional expressions of the leader may sometimes also evoke perceptions of honesty and credibility (Bucy, 2000; Bono and Ilies, 2006) and perceptions of status (Tiedens, 2001). Expression of negative emotions may also be a relieving experience; display rules of emotion and impression management may lead to positive effects on organizational level, but they may result to emotional labor for the individual (e.g., Grandey, 2000). It is possible that in the current study the emotionally intelligent leaders suppressed negative emotional expressions not only because it was emotionally intelligent in the current situation, but also because of (organizational and/or national) cultural display rules encouraged this. It must be noted, however, that not all negative emotions evoke similar effect during a feedback situation. A study by Johnson and Connelly (2014) showed that an informal feedback in angry tone prompted reciprocal anger whereas feedback with a disappointed tone prompted feelings of guilt in the receiver.

The observed results of the current study encourage to continue extending previous study of EI in negotiation context (e.g., Der Foo et al., 2004) also to other work life interactions and to study particular forms of emotional expressions, as suggested by Morris and Keltner (2000).

### Strengths of the Current Study

Many of the previous studies on leader’s social interaction have used controlled settings, and students acting as leaders and subordinates. One apparent strength of the current study is the natural setting with actual leader–follower dyads that had an actual performance review discussion, or a semi-structured dialog on each partner’s areas of development.

There are a few previous studies where the EEG frontal asymmetry has been related to individual differences in emotional processing (e.g., Coan and Allen, 2004; Mathersul et al., 2008) and EI (e.g., Craig et al., 2009). However, there are not many studies that would have used the EEG during naturalistic interaction. Another strength of the current study is the dyadic analysis method, which allowed the study of the actor and partner effects of the trait EI on the physiological activity and self-reported emotions during the interaction and thus contributing to the understanding of the biological correlates of trait EI.

### Limitations of the Current Study

There are obvious problems with the self-report method in general, especially related to the social desirability bias in responding. These problems may be highlighted in organizational settings, where, in general, it is important to give a competent perception of oneself. However, in Study 1 a 360-method for assessing trait EI was used, and both of the studies provided converging results regarding the positive effects of trait EI during social interaction. Although, it must be noted that the used 360-scale is currently under development and would need to be validated with a larger sample.

The obtained result could, at least partly, be explained also by the high trait EI leaders being more able to pick for the discussion followers that viewed them as favorably. That is, in addition to high trait EI leaders having better relations in general with their followers, and also them being better in handling critical leadership situations (such as a review discussion), it is possible that they used their high trait EI in detecting the most suitable subordinates for the discussion. This may have led to overly positive discussions where negative topics were avoided. Of course, the subordinate had to be willing to participate to the measurements as well, and it is possible that a quarrelsome subordinate would have declined to participate. It would have been difficult to avoid these effects with the current setting. However, the managers were asked not to select their “best friends” as a partner for the setting, but to preferably choose someone whom they felt that they had something to develop with in their leader–follower relationship.

### Practical Implications

There are various studies that have documented the positive effects of EI training of managers (e.g., Slaski and Cartwright, 2003; Groves et al., 2008; for a review, see Riggio and Lee, 2007). Emotional expressivity is a skill set that can be trained and developed just like the other emotional skills. The study by Towler (2003) showed the positive effects of charismatic influence training, including skills such as emotional expressivity, on influencing the followers. Charismatic influence training had a stronger effect than more traditional coaching of presentation skills. Video recordings or interactions are especially beneficial in training of emotional expressivity, since non-verbal expressions of emotion may be subtle and difficult to detect consciously (Riggio and Reichard, 2008). Adding visualizations of physiological activation (e.g., facial EMG responses) of both participants to the video could possibly be a fruitful material for training programs of emotional expressivity. Similarly, the 360-feedback on the manager’s actual expressivity would provide tangible development tasks.

### Future Directions

Given the effects that positive emotions have on the group functioning (e.g., Sy et al., 2005), the facilitative effects of EI to positive emotional contagion in work groups is suggested as one promising future direction of research. Chang et al. (2011) showed that both average member EI and group leader’s EI have positive effects on group trust and performance. Menges (2012) conceptualizes organizational EI that could be developed by providing the individuals with EI training. In the study of group or organizational EI, also psychophysiological methods could be used. For example, in a study by Henning et al. (2009) social psychophysiological compliance, operationalized as heart rate variability synchronization, was found to be related to effectiveness in a team of four researchers during actual meetings. Similarly, physiological indexes related to emotional processing and expressivity could be monitored in a team.

## Author Contributions

MS planned and conducted the data collection and analyses, and wrote the manuscript. NR participated to the planning of the data collection and analyses, and to the writing of the manuscript.

## Conflict of Interest Statement

The authors declare that the research was conducted in the absence of any commercial or financial relationships that could be construed as a potential conflict of interest.
